# Editorial: Humans in an Animal's World—How Non-human Animals Perceive and Interact With Humans

**DOI:** 10.3389/fpsyg.2021.733430

**Published:** 2021-09-01

**Authors:** Christian Nawroth, Luigi Baciadonna, Nathan J. Emery

**Affiliations:** ^1^Research Institute for Farm Animal Biology, Institute of Behavioural Physiology, Dummerstorf, Germany; ^2^Department of Life Sciences and Systems Biology, University of Turin, Turin, Italy; ^3^Biological and Experimental Psychology, School of Biological and Chemical Sciences, Queen Mary University of London, London, United Kingdom

**Keywords:** animal cognition, animal welfare, conservation biology, heterospecific communication, human-animal interaction

Whilst humans undisputedly shape and transform most of earth's habitats, the number of animals (captive and wild) living on this planet far outnumbers that of humans. Humans, therefore, inevitably interact with different animals in a variety of contexts: we keep them for companionship, farm them for their products, use them for biomedical research, house them in zoos and sanctuaries, and interact with animals in the wild. How humans engage in these interactions has been extensively debated in areas such as ethics, sociology, and psychology. Given the rise of animal welfare concerns over the last decades, but also our growing interest in understanding the minds of non-human animals, there is now a strong demand to shift from a rather traditional anthropocentric view and focus on how animals themselves perceive and interact with humans in this variety of contexts. Over a range of fields, such as psychology, ethology and animal welfare science, questions on how non-human animals acquire knowledge about humans, how this knowledge is generalised and how it can spread socially are of increasing relevance to mediating conflicts arising from human-animal interactions across different settings (Bensky et al., 2013; Nawroth et al., 2019). This is the focus of this *Frontiers* Research Topic.

This Research Topic comprises 18 articles, including state-of-the-art empirical work as well as review articles concerning the role of humans in the sensory and cognitive world of non-human animals, either in captivity or in the wild. It provides discussions on the applied implementation of these findings (e.g., for conservation attempts or farmed animal husbandry management) and considerations of future interdisciplinary approaches and applications.

## Companion Animals

The Research Topic attracted many papers examining the cognitive and perceptual abilities of companion animals. This is no surprise given their popularity and immersion in our everyday lives. Dogs, for example, have shared a long journey with humans (Davis and Valla, 1978) and, throughout the domestication process, their communicative abilities to interact with humans have been profoundly affected. In the past, most of the research effort in this area was directed toward humans' understanding of dog behaviours, whereas a new trend emerged in which the focal point is to explore what dogs understand about human behaviours.

The relationship that humans have with their pet dogs is often quite intense, leading to a strong bond with the owner, caregiver, or extended family, including children. Benz-Schwarzburg et al. discuss the nature and ethical dimension of these bonds in the light of current scientific knowledge on the social skills of dogs. The focal point of their review considers human-dog interactions from the perspective of the dog, with the ultimate goal to inform human actions and identify responsibility toward their “best friend.” Koyasu et al. reviewed the communication between humans and dogs and humans and cats. Although both species followed different domestication trajectories, both dogs and cats are able to communicate non-verbally with humans. The authors specifically focus on their gazing behaviour which is an important signal for humans, describe the communicative function of dogs' and cats' eye-gaze behaviour with humans, and present a research-based approach to multimodal interactions between dogs/cats and humans.

Interactions initiated by dogs toward humans are a crucial part in the human-dog interplay. For example, the expression of so-called “Puppy Dog Eyes” (i.e., raising of the eye brow) has been suggested to be sensitive to the attentive stance of humans and might thus imply a possible communicative function. However, Bremhorst et al. showed that this expression was more often shown in non-social, rather than social contexts–thus challenging its communicative function, suggesting an association with eye movements as an alternative explanation for its expression. Dogs also often look back to humans when they are confronted with a difficult problem they perceive as unsolvable. Some claim that this might be an indication of decreased problem-oriented behaviour, whilst others interpret this as a stronger motivation to interact with humans in general. To find out whether specific training, such as actively helping people as assistance and therapy dogs, increases problem-oriented behaviour, Carballo et al. compared the behaviour of dogs with different training experiences. They showed that training, and specifically training to help people, led to increased problem-oriented behaviour, and in turn to less human-directed behaviour in an unsolvable task.

The ability of dogs and other companion animals to interact and communicate with humans might also affect therapeutic contexts that cover both humans and companion animals. Grandgeorge et al. explored the pattern of visual attention during dog-child and cat-child interactions in children with typical development and in children suffering from autism spectrum disorders (ASD). While dogs displayed more gazes, cats showed glances, which could be considered more subtle, toward humans. Children with ASD preferentially directed their visual attention toward their pet cat, but the amount of visual attention toward pet cats and pet dogs was similar for the children with typical development. The authors proposed that ASD children perceived their cats' repeated glances less invasively and more comfortably than those of their dogs. This might likely increase the chances for ADS children to develop a bond with their pet cat compared to their pet dogs. Wanser et al. provided evidence that dogs with a secure attachment, measured by the Secure Base Test (Ainsworth et al., 1978) within the context of an Animal Assisted Intervention, have the potential to change the overall attachment style between a family dog and a child to a more secure attachment. For example, dogs with a strong attachment to the parents developed also a more secure attachment to the family's child during the intervention.

Globally, free-ranging dogs constitute the majority of domesticated dogs under direct humans' supervision (Hughes and Macdonald, 2013; Lord et al., 2013) and in some parts of the world the presence of stray dogs living close to urbanised areas presents a challenge because of potential conflicts with the local community. Bhattacharjee and Bhadra examined intraspecific (dog-dog) and interspecific (dog-human) interactions in twelve groups of free ranging dogs living in intermediate and high level areas of human activity, using social network analysis. The analysis revealed that the frequency of interspecific interactions was higher than intraspecific interactions, regardless of the urbanised living condition; humans were the main initiators of positive and negative interactions with the stray dogs. A better understanding of the interactions between stray dogs and humans can thus help to address the concerns generated by stray dogs living in urbanised areas.

Many of the communicative capacities of companion animals toward humans have been proposed to be affected by domestication. In particular, the selection for tameness has been proposed as the primary mechanism of domestication and has also been associated with changes in autonomic nervous system regulation. Jean-Joseph et al. aimed to test dogs and wolves in different activity contexts, either alone, with a human or with a conspecific. Although the authors found context-specific differences between dogs and wolves, e.g., dogs were more relaxed than wolves when at rest and close to a familiar human, no general differences between the two groups emerged suggesting that the impact of selection for tameness on the modulation of the autonomic nervous system is more complex than previously thought.

## Farmed Animals

The relationship between humans and farmed animals is under special scrutiny. Farmed animals are kept for production purposes, and economic incentives can often be detrimental to a good human-animal relationship, subsequently leading to diminished animal welfare in general. Although a good human-animal relationship cannot alone ensure good welfare to farm animals, it is a crucial aspect to enhancing their quality of life. Rault et al. review the mechanisms underlying this relationship and particularly highlight the need for reliable indicators for this relationship as assessing the quality of human-animal interactions can be challenging. Crucially, the authors also offer perspectives on how to refine those indicators.

In the relationship between farmed animals and humans, tactile perception, such as gentle stroking, plays a crucial role in decreasing stress, and fearfulness (Hemsworth, 2003; Tallet et al., 2014). Lange et al. refined this approach by investigating whether the perception of human voices, either live or via recordings, during these interactions could affect cows' emotional experience. Their findings suggest that live talking was pleasurable to the animals and had a stronger relaxing effect than voice recordings. Specific details of routine management may also have an impact on the relationship between farmed animals and humans. Aigueperse and Vasseur showed that provision of an outdoor exercise area can affect cows' reactivity toward humans. The authors found seasonal differences of this effect, which they linked to different handling styles over the seasons. That means that the way cows are handled during these events provides opportunities to facilitate future handling.

Beyond being perceived as the individuals that handle animals, humans might also provide a form of enrichment to animals with their mere presence. Villain et al. followed-up on this idea and investigated whether the response of pigs to an inanimate manipulable object and a familiar human differs. After a brief period of isolation, pigs were reunited with either the object or the human. Only the reunion with the human led to the production of positive shorter grunts, usually associated with positive situations, leading the authors to suggest that positive pseudo-social interactions with a human could help to enrich pigs' environment.

More subtle cues from humans, such as gaze direction, can also be perceived by domestic animals. Although previous studies on gaze following were primarily conducted on primates and canids, there has been a recent trend to test more uncommon taxa, including ungulates, in order to identify the evolutionary pressures leading to the emergence of gaze following skills. Schaffer et al. provided experimental evidence of gaze following skills in domestic, but also non-domestic ungulates, highlighting that selection pressures caused by domestication might not be necessary to follow human gaze.

Interactions with humans can also have a profound effect on physiological parameters of farmed animals. Scopa et al. presented a technical study assessing the cardiac activity of horses when they interact with humans. The horses were more relaxed when being physically touched by a familiar handler, as compared to unfamiliar humans. Interactions between humans and farmed animals are almost always not neutral–so these situations can have a strong positive, but also negative impact at the physiological and behavioural level. Studies on interactions between humans and farmed animals may therefore provide practical suggestions on how humans should interact and manage such animals.

## Wild Animals

One aspect of human-animal interactions that has received relatively less attention so far are interactions of non-domestic animals with humans, for example in the wild or in a zoo setting. These animals occupy important niches in anthropogenic environments, and future research should focus on how human activity and behaviour may affect their welfare and how to solve conflicts of this cohabitation (especially in animals living in the wild).

Anthropogenic activity has profoundly changed ecosystems and often brought humans and wild animals into close proximity, and occasionally conflict. Therefore, skills such as recognising humans could be particularly advantageous for wild animals as they might enable them to access resources or avoid potentially negative consequences. Goumas et al. review how wild animals modulate their responses toward humans by also describing the most likely cognitive processes involved. In addition, they also discuss how certain cognitive abilities might be under indirect human selection and argue about its potential impact on the wild population. They conclude that future research should aim to better understand these dynamics and inform adequate conservation policies and wildlife management.

Blum et al. investigated the ability to differentiate between humans in captive ravens. In their study, common ravens quickly distinguished between a dangerous human (carrying a dead raven) vs. a non-threatening human. The ravens were still responding to the potentially dangerous human after 4 years without any further associations. Considering that ravens exploit human resources but do not live in highly urbanised areas, they represent a valuable model species to investigate which cognitive mechanisms are involved in individual human recognition. Some wild animals, such as elephants, have a long history of living alongside humans, although they were never domesticated. This context provides an interesting opportunity to test animals for socio-cognitive skills that have largely been investigated in domestic animals, such as dogs (Kaminski and Nitzschner, 2013). Here, Jim et al. investigated whether Asian elephants can form a reputation about humans using direct or indirect experience (e.g., eavesdropping) of human interactions. Their results suggest that when elephants can choose between a cooperative and a non-cooperative human they choose indifferently and the authors discuss potential issues linked with the sample size and methodological details. In particular, they suggest taking species-specific sensory-perceptual abilities into account (Plotnik et al., 2014), especially when the tasks involve interactions with other species.

In a zoo setting, visitors can affect the behaviour of captive wild animals. This so-called “visitor effect” has received scientific attention for the potentially negative impact it can have on animals kept in zoos. However, quite often other factors that are related to visitor activity might lead to an overestimation of the impact of visitors *per se*. In this context, Rose et al. studied the behaviour of hornbills in a zoo setting and found no general visitor effect. The authors here show the necessity to integrate climatic conditions, the sex of the animal and the number of visitors on the behavioural parameters analysed to gain a more complete picture on how visitors might impact on the welfare of zoo animals.

In conclusion, the contributions to this Research Topic expand our understanding of how animals in different contexts and with different life histories perceive and interact with humans, raising new possibilities for mitigating problems where the interests of humans and animals are in conflict with each other.

## Author Contributions

All authors listed have made a substantial, direct and intellectual contribution to the work, and approved it for publication.

## Conflict of Interest

The authors declare that the research was conducted in the absence of any commercial or financial relationships that could be construed as a potential conflict of interest.

## Publisher's Note

All claims expressed in this article are solely those of the authors and do not necessarily represent those of their affiliated organizations, or those of the publisher, the editors and the reviewers. Any product that may be evaluated in this article, or claim that may be made by its manufacturer, is not guaranteed or endorsed by the publisher.
